# Illicit Stimulant Use among Medical Students in Riyadh, Saudi Arabia

**DOI:** 10.7759/cureus.6688

**Published:** 2020-01-17

**Authors:** Feras A Alrakaf, Faris H Binyousef, Abdulaziz F Altammami, Ahmed A Alharbi, Asem Shadid, Nader Alrahili

**Affiliations:** 1 Medicine, Imam Mohammad Ibn Saud Islamic University, Riyadh, SAU; 2 Psychiatry, Imam Mohammad Ibn Saud Islamic University, Riyadh, SAU; 3 Psychiatry, Imam Mohammad Ibn Saud Islamic University, Riyadh , SAU

**Keywords:** stimulants, attention-deficit hyperactivity disorder (adhd), medical students, riyadh, academic performance, illicit use, abuse, motives

## Abstract

This study aims to assess the prevalence of illicit use of stimulants and attention deficit hyperactivity disorder (ADHD) among a sample of medical students at the main universities in Riyadh, Saudi Arabia and their motivation for use. We examine the association between the use of stimulants and the students' academic performance. We also look into the possible adverse consequences of illicit stimulant use among students.

The competitive nature of medical school might place the students at a higher risk of using stimulant drugs illicitly. Acquiring these stimulants illegally has become easier since the diagnosis and treatment of ADHD have risen. We are unaware of any other study exploring the prevalence of and motivation for illicit use of stimulants among medical students in Riyadh.

A cross-sectional web-based survey was the study design we chose as we were targeting medical students in three governmental medical colleges in Riyadh, Saudi Arabia. The total sample population of 1,177 participants was divided into the three following groups: no previous use of stimulant drugs (Group 1), illicit use (Group 2), and medical use (Group 3). Of the 1,177 medical students, 29 (2.46%) were found to be using stimulants illicitly; 39 (3.31%) were using the stimulants medically as they had been diagnosed with ADHD. The ability to prolong study time was reported as the most common motive for illicit use by many students.

The present study contributes to the literature by casting light on this serious issue in Riyadh. More educational effort is needed to promote awareness about the adverse effects of ADHD drugs and their illicit use among students.

## Introduction

Illicit use of stimulants has been a major public health issue [1]. Illegal access to the stimulants has become easier since the diagnosis and treatment of attention deficit hyperactivity disorder (ADHD) have risen in recent times [2]. According to Matthews et al., ADHD is “a prevalent and persistent psychiatric disorder that emerges early in childhood, with a current prevalence rate of 5% in children 4-17-years old. The disorder is classically characterized by symptoms of inattention, impulsivity, and hyperactivity” [3]. Various drugs have been used in the management of ADHD, such as Adderall (mixed salts amphetamine), Dexedrine (dextroamphetamine), and Ritalin (methylphenidate). A cross-sectional web-based survey was performed at one public and one private university in the southeastern US. The sample included 3,400 undergraduate college students from these two different universities. Among the participants, 5.4% of students had been found to be using nonmedical ADHD drugs for the past six months [4].

The competitive nature of medical school might place the students at a higher risk of using stimulant drugs illicitly. A previous study showed that even an ordinary college student is at a higher risk compared with peers of the same age who are not attending college (5.7% versus 2.5%, respectively) [5]. While stimulant drugs are highly effective for treating ADHD, they have multiple risk factors that negatively affect the illicit users [1]. We are unaware of any existing study that examines the prevalence and motivation for illicit use of stimulants among medical students in Riyadh. We take this to indicate that we need to take this issue more seriously.

Through this study, we aspire to make healthcare providers more aware of this issue when dealing with individuals with ADHD and to factor in the illicit use of these psychostimulants since they have both positive and negative effects. Healthcare providers must work on strategies to detect and minimize psychostimulant abuse. Since there are no available data about the illicit use of stimulants in Riyadh, Saudi Arabia, most universities do not have a specialist in this field on-hand to educate students on the adverse consequences of using stimulant drugs illicitly. And we believe that having such specialists would help reduce the prevalence of stimulant abuse. After learning the most frequent motives reported by students, we can look for solutions that would provide the students with their desired outcomes without the illicit use of stimulant drugs. A study that focused on the motives of using these stimulants sampled 9,161 undergraduate college students randomly [6]. The most prevalent motive found was the ability to concentrate more that these stimulants reportedly provided (58%). The other most prevalent motives were the roles the stimulants reportedly played in increasing alertness and helping to get high (43%) [6].

This study aimed to determine several things. First, we intended to determine the prevalence of illicit use of stimulants and the prevalence of ADHD among a sample of medical students at the main governmental universities in Riyadh. Secondly, we tried to assess the students’ motivation for the use of stimulants. Lastly, we wanted to achieve a better understanding of the possible adverse consequences of illicit stimulant use in various spheres, including academic performance.

## Materials and methods

Study design

We conducted a cross-sectional study that used an online questionnaire distributed by email and phone among all the medical students of the three government medical colleges in Riyadh, Saudi Arabia (Imam Mohammed bin Saud University, King Saud University, and King Saud bin Abdulaziz University for Health Sciences). All participants provided informed consent and were ensured privacy; all their answers would be secure and handled only by the research team. SurveyMonkey cloud-based software (SVMK Inc., San Mateo, CA) was used to collect the participant’s responses. Repeated submissions from the same participants were weeded out by linking each response with their IP address via the SurveyMonkey website.

The participants were given three weeks to complete the questionnaire. Of a total of around 3,550 medical students, 1,177 students completed the study, indicating a response rate of 33.15%. The participants were classified into one of three groups: no previous use of stimulant drugs (Group 1), illicit use (Group 2), and medical use (Group 3).

Study method

The questionnaire was designed to collect demographic information including age, gender, year of study, university, academic performance [grade point average (GPA)], average family monthly income, and smoking status. The collected data also included marital status, average hours of sleep, and the number of study days missed during the previous month. Since one of our objectives was to identify the prevalence of ADHD among medical students, we asked the respondents if they had been diagnosed with ADHD, and if they were taking stimulant drugs for this condition. A brief introduction about the stimulants and their use was provided to ensure that all the students had a basic knowledge, which would allow them to answer the questions properly.

To achieve our main objective of exploring illicit use of stimulants, the following question was asked: “Have you ever used stimulants such as Fenethylline, Ritalin, Concerta, Dexedrine, Adderall, or Vyvanse?”. Based on the response to this question, the students were divided into the three groups mentioned above. Those students who reported illicit use were asked further questions.

To estimate the frequency of illicit use, three questions were asked. The first question was: “On how many occasions in the last year (if any) have you taken amphetamines on your own-that is, without a doctor telling you to take them?” [6]. The second question was: “On how many occasions in your time in medical school (if any) have you taken amphetamines on your own-that is, without a doctor telling you to take them?”.The final question was: “In which year of medical school did you first use stimulants illicitly?”.

Three additional questions regarding drug use were asked. The first question: “What is the quantity you use per occasion?” [7]. Secondly, we asked about the route of administration of the drugs [7]. The third question explored the possible sources for the stimulants: “Where did you get the amphetamines you used without a doctor’s orders?”. Many possible answers were listed for this question, such as that they were provided for free by a relative, bought from a drug dealer/stranger, and taken from a friend without asking, etc [8].

We hoped to identify the reason for taking the stimulants via this question: “What have been the most important reasons for you to take stimulants without doctor’s orders?”. A variety of possible motives were listed including the main motive we were expecting to see (i.e., to help with academic performance) along with other non-academic motives, such as to have a good time with friends, to help lose weight, to achieve euphoria, etc [6]. To know if the students achieved the motive they desired, a follow-up question was asked: “Is taking the ADHD medication producing the results desired?” [4]. The following question focused on the effect of the stimulants on academic performance, irrespective of whether the students took it for academic reasons or not. We assessed the changes in academic performance (GPA) via this question: “Has there been any change observed in your GPA since you used the stimulants?” [9].

Finally, we wanted to explore the side effects of using these stimulants primarily by comparing medical users and illicit users; we also wanted to identify the frequency of each side effect. This information was obtained by the following question: “Have you experienced any of the listed adverse effects while using the stimulants? (mark all that apply).” The possible adverse effects listed were "headaches, stomach aches, irritability, sadness, reduced appetite, sleep difficulty, dizziness, difficulty getting along with friends, becoming dependent on ADHD medication", and "Other" [4].

Statistical analysis

The statistical analysis was performed using RStudio version 1.1.363 (RStudio Inc., Boston, MA). Categorical data were summarized using counts and percentages. Chi-square test of independence (or Fisher’s exact test when appropriate) was used to assess whether the distribution of demographic factors across the three groups was significantly different from what was expected under the null hypothesis. Hypothesis testing was performed at the .05 level of significance. Bar and pie charts were used to visualize the results.

## Results

Descriptive statistics

**Figure 1:**
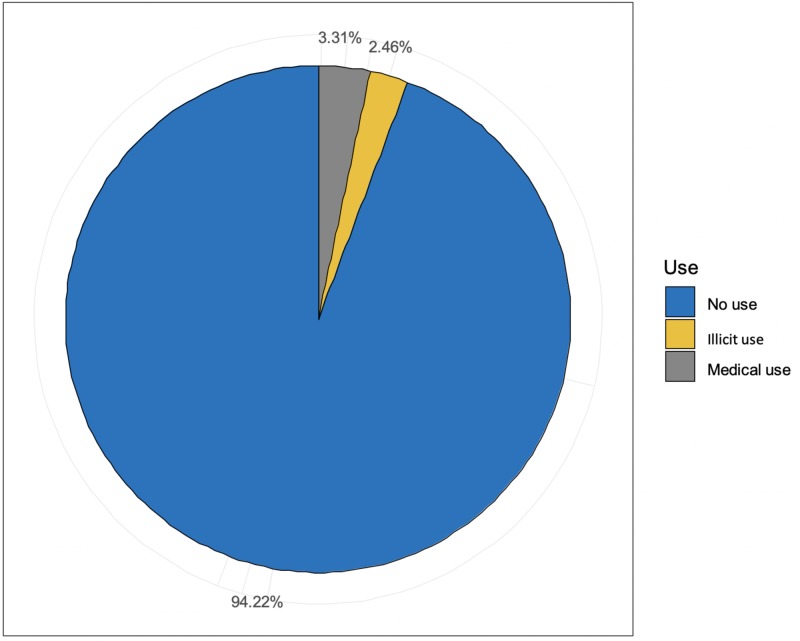
Stimulant use across the study sample

The study sample included 1,177 medical students. Stimulants (medically and illicitly) were found to be used by 68 (5.8%) participants. The medical use of stimulants was reported by 39 (57.4%) of the students who admitted to using stimulants. The illicit use of stimulants was reported by the remaining 29 students (42.6%). The overall percentages are shown in Figure 1. The demographic characteristics of the study sample are shown in Table 1.

**Table 1:** Demographic characteristics of the study sample N: number of students; SAR: Saudi riyal

	N (%)
Gender	
Female	576 (48.9%)
Male	601 (51.1%)
Age, years	
18–20	400 (34.0%)
2123	565 (48.0%)
24 and older	212 (18.0%)
Marital status	
Single	1066 (90.6%)
Married	111 (9.43%)
Average monthly income, SAR	
Less than 5,000	50 (4.25%)
5,000–10,000	131 (11.1%)
10,000–15,000	133 (11.3%)
15,000–20,000	215 (18.3%)
More than 20,000	648 (55.1%)

Men and women were equally represented in the study sample (51.1% and 48.9%, respectively). Almost half of the participants were 21-23 years of age (n = 565, 48%). The 18-20-year-old (n = 400, 34%) and ≥24 years (n = 212, 18%) age groups were also well represented in the study sample. Most of the study participants were single (n = 1,066, 90.6%) while some were married (n = 111, 9.43%). More than half of the participants reported an average monthly income greater than 20,000 Saudi riyals (SAR, n = 648, 55.1%).

Each of the three universities included, as well as the academic class years of the students, were equally represented in the study sample (Table 2). The self-reported GPA was 4.5-5 by 45.4% (n = 534) of students. Some participants (n = 163, 13.8%) reported failing one to three blocks or courses and only 59 (5.01%) reported failing more than three courses. The remaining students (n = 955, 81.1%) had not failed any block or course. More than half of the participants reported an average sleeping time of six to eight hours (n = 615, 52.3%). Roughly one-quarter of the study participants reported smoking (n = 261, 22.2%). More than half of the students who reported using stimulants (n = 68) replied “yes” when asked whether they had ADHD or not (n = 39, 57.4%). More than 60% of the students reported missing none to three days during the last four weeks (n = 747, 63.5%), and only 121 (10.3%) reported missing more than 10 days.

**Table 2:** Study-related characteristics of the cohort N: number of students; GPA: grade point average; ADHD: attention deficit hyperactivity disorder

	N (%)
University	
‎King Saud Bin Abdulaziz University for Health Sciences	408 (34.7%)
Imam Mohammed bin Saud university	412 (35.0%)
King Saud University	357 (30.3%)
Class year	
First	251 (21.3%)
Second	240 (20.4%)
Third	235 (20.0%)
Fourth	186 (15.8%)
Fifth	265 (22.5%)
GPA	
Less than 3	56 (4.76%)
3–3.5	88 (7.48%)
3.5–4	179 (15.2%)
4–4.5	320 (27.2%)
4.5–5	534 (45.4%)
Ever failed a block or course	
No	955 (81.1%)
1–3	163 (13.8%)
More than 3	59 (5.01%)
Missed days during the last four weeks	
0–3 days	747 (63.5%)
4–6 days	204 (17.3%)
7–10 days	105 (8.92%)
More than 10 days	121 (10.3%)
Average sleeping hours	
Less than 3 hours	45 (3.82%)
4–5 hours	372 (31.6%)
6–8 hours	615 (52.3%)
More than 8 hours	145 (12.3%)
Smoker	
No	916 (77.8%)
Yes	261 (22.2%)
ADHD (n = 68)	
No	29 (42.6%)
Yes	39 (57.4%)

Factors associated with illicit use 

The distribution of gender across the three groups (n = 1,177) was not significantly different from what was expected under the null hypothesis (p: >.05). However, the distribution of the remaining demographic characteristics was significantly different from what was expected across the three groups. The percentage of medical students who were 24 years of age or older was lower in Group 1 compared to Groups 2 and 3 (p: <.001). Similarly, the percentage of married individuals was significantly higher in Groups 2 and 3 compared to Group 1 (p: <.001). Medical students who reported having a monthly income greater than 20,000 SAR was higher in Group 1 (n = 631, 56.9%) compared to Groups 2 (n = 11, 37.9%) and 3 (n = 6, 15.4%). The distribution of monthly income categories across the three groups was significantly different from what was expected under the null hypothesis (p: <.001). (Table 3).

**Table 3:** Association between demographic factors and stimulant use SAR: Saudi riyal *statistically significant

	No use	Illicit use	Medical use	P-value
	N = 1,109	N = 29	N = 39	
Gender				.754
Female	540 (48.7%)	16 (55.2%)	20 (51.3%)	
Male	569 (51.3%)	13 (44.8%)	19 (48.7%)	
Age, years				.001
18–20	393 (35.4%)	2 (6.90%)	5 (12.8%)	
21–23	543 (49.0%)	12 (41.4%)	10 (25.6%)	
24 and above	173 (15.6%)	15 (51.7%)	24 (61.5%)	
Marital status				.001
Single	1,030 (92.9%)	17 (58.6%)	19 (48.7%)	
Married	79 (7.12%)	12 (41.4%)	20 (51.3%)	
Monthly income, SAR				.001
Less than 5,000	45 (4.06%)	1 (3.45%)	4 (10.3%)	
5,000–10,000	114 (10.3%)	8 (27.6%)	9 (23.1%)	
10,000–15,000	122 (11.0%)	3 (10.3%)	8 (20.5%)	
15,000–20,000	197 (17.8%)	6 (20.7%)	12 (30.8%)	
More than 20,000	631 (56.9%)	11 (37.9%)	6 (15.4%)	

**Figure 2:**
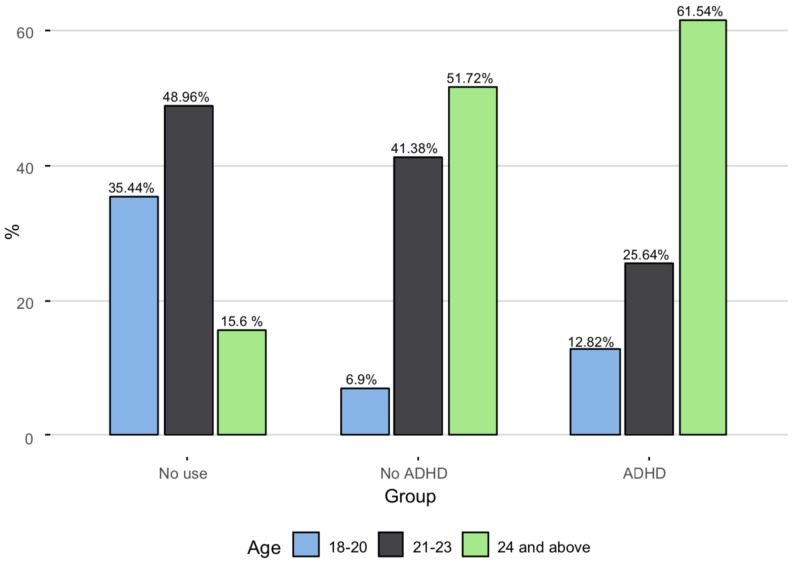
Distribution of age across the three groups ADHD: attention deficit hyperactivity disorder The Y-axis represents the percentage of the respondents

The percentage of older students (24+ years) was higher in Groups 2 and 3 compared to Group 1. (Figure 2)

**Figure 3:**
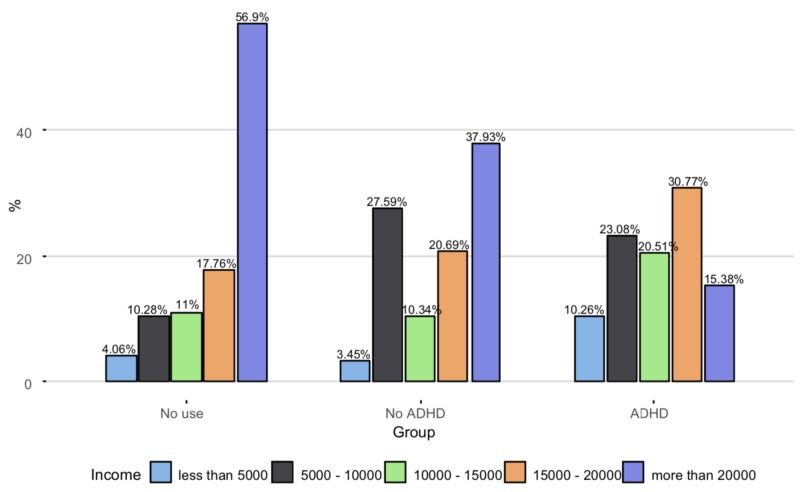
Distribution of income categories across the three groups ADHD: attention deficit hyperactivity disorder The Y-axis represents the percentage of the respondents

The percentage of participants who reported high income (>20,000 SAR per month) was higher in Group 1 compared to Groups 2 and 3, explaining the statistically significant chi-square test results. (Figure 3).

Table 4 shows the association between study-related factors and stimulant use across the three groups. The distribution of universities across the three groups was not significantly different from what was expected under the null hypothesis (p: >.05). The distribution of class year across the three groups was significantly different from what was expected under the null hypothesis (p: <.001). The percentage of fifth-year students was lower across participants who did not report using stimulants compared to the remaining two groups (19.9%, 58.6%, and 69.2%, respectively). The distribution of GPA was also different across the three groups (p: <.001). Higher GPA (4.5-5) was observed across participants who did not use stimulant drugs (47.7% in Group 1 vs. 17.2% and 0% in Groups 2 and 3, respectively; p: <.001), indicating that students with high GPA are less likely to report illicit use. Students who did not use stimulants were less likely to report more than 10 missed days during the previous month (p: <.001). The percentage was lower in Group 1 (n = 102, 9.2%) compared to Groups 2 (n = 6, 20.7%) and 3 (n = 13, 33.3%). This indicates that illicit use is associated with the number of missed days during the previous month. Participants who reported average sleeping hours greater than 8 hours were more common in Group 2 and 3 compared to Group 1 (p: <.001). The prevalence of smoking was also higher in Groups 2 (n = 23, 79.3%) and 3 (n = 31, 79.5%) compared to group 1 (n = 207, 18.7%). These results indicate that most of the study-related factors are associated with illicit stimulant use.

**Table 4:** Association between study-related factors and stimulant use GPA: grade point average

	No use	Illicit use	Medical use	P-value
	N = 1,109	N = 29	N = 39	
University				.862
King Saud Bin Abdulaziz University for Health Sciences	382 (34.4%)	12 (41.4%)	14 (35.9%)	
Imam Mohammed bin Saud University	389 (35.1%)	8 (27.6%)	15 (38.5%)	
King Saud University	338 (30.5%)	9 (31.0%)	10 (25.6%)	
Class year				.001
First	247 (22.3%)	2 (6.90%)	2 (5.13%)	
Second	237 (21.4%)	1 (3.45%)	2 (5.13%)	
Third	227 (20.5%)	3 (10.3%)	5 (12.8%)	
Fourth	177 (16.0%)	6 (20.7%)	3 (7.69%)	
Fifth	221 (19.9%)	17 (58.6%)	27 (69.2%)	
GPA				.001
Less than 3	43 (3.88%)	6 (20.7%)	7 (17.9%)	
3–3.5	73 (6.58%)	6 (20.7%)	9 (23.1%)	
3.5–4	164 (14.8%)	5 (17.2%)	10 (25.6%)	
4–4.5	300 (27.1%)	7 (24.1%)	13 (33.3%)	
4.5–5	529 (47.7%)	5 (17.2%)	0 (0.00%)	
Ever failed a block or course				.001
No	934 (84.2%)	11 (37.9%)	10 (25.6%)	
1–3	140 (12.6%)	10 (34.5%)	13 (33.3%)	
More than 3	35 (3.16%)	8 (27.6%)	16 (41.0%)	
Missed days during the last four weeks				.001
0–3 days	726 (65.5%)	14 (48.3%)	7 (17.9%)	
4–6 days	189 (17.0%)	5 (17.2%)	10 (25.6%)	
7–10 days	92 (8.30%)	4 (13.8%)	9 (23.1%)	
More than 10 days	102 (9.20%)	6 (20.7%)	13 (33.3%)	
Average sleeping hours				.001
Less than 3 hours	35 (3.16%)	2 (6.90%)	8 (20.5%)	
4–5 hours	356 (32.1%)	6 (20.7%)	10 (25.6%)	
68 hours	592 (53.4%)	12 (41.4%)	11 (28.2%)	
More than 8 hours	126 (11.4%)	9 (31.0%)	10 (25.6%)	
Smoker				.001
No	902 (81.3%)	6 (20.7%)	8 (20.5%)	
Yes	207 (18.7%)	23 (79.3%)	31 (79.5%)	

Other Characteristics of participants who use stimulant medications (n = 68)

**Figure 4:**
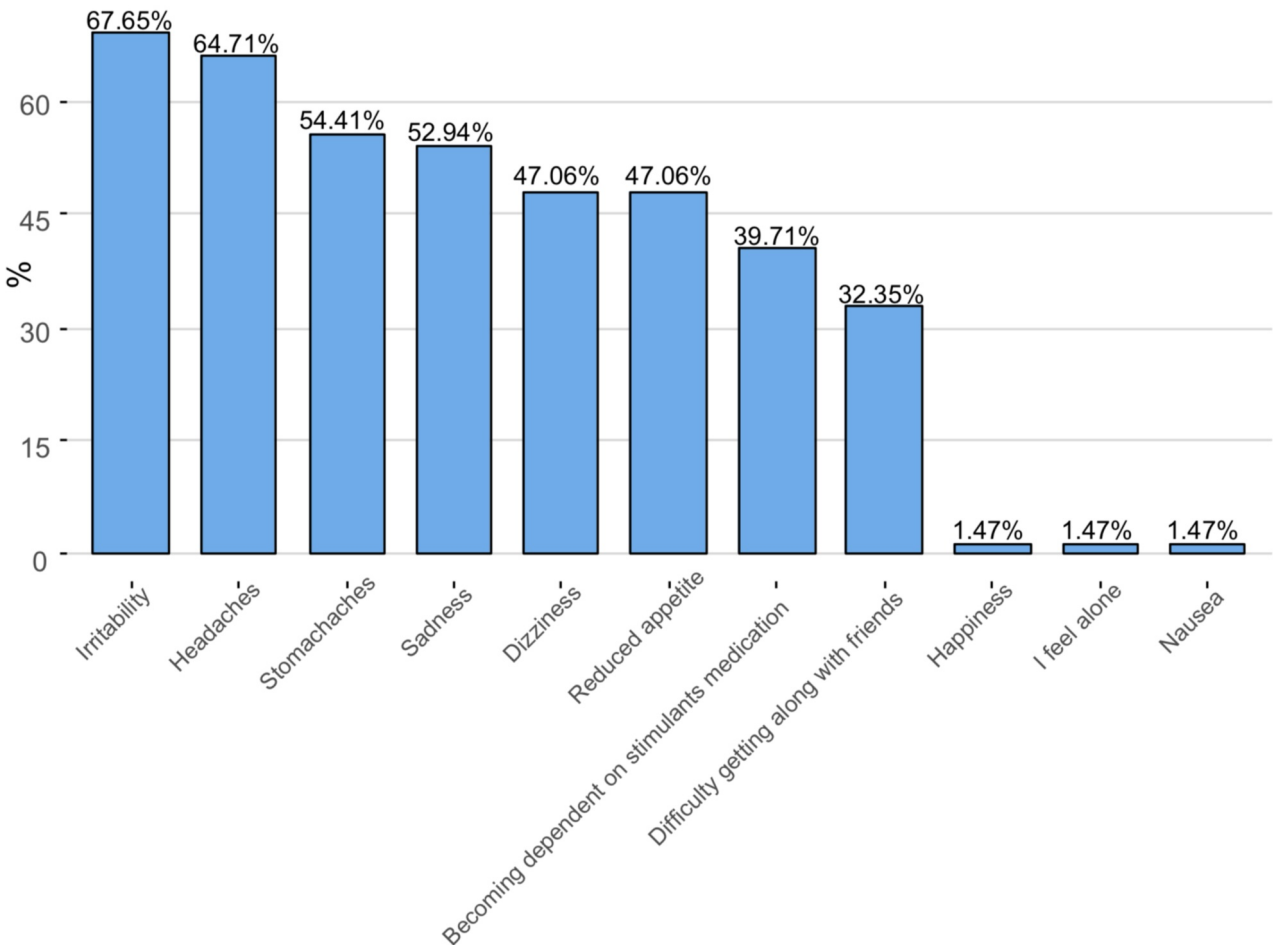
Reported adverse effects by users of stimulant drugs The Y-axis represents the percentage of stimulant users

Irritability and headaches were the two most recorded adverse effects (67.65% and 64.71% respectively), whereas loneliness, nausea, and euphoria where the least noted adverse effects (1.47% for each), as shown in Figure 4.

 

D.Characteristics of participants who reported illicit use of stimulants (n = 29)

The percentage of students that started using stimulants illicitly in the first (n = 5, 17.2%), second (n = 9, 31%), and third (n = 8, 27.6%) years was higher compared to the percentage that started doing so in the fourth (n = 4, 13.8%) and fifth (n = 3, 10.3%) years. Nearly half of the students who reported illicit use mentioned that taking stimulants produced the desired results (n = 14, 48.3%). Regarding the observed changes in GPA, 41.4% (n = 12) of the students who reported illicit use mentioned that their GPA had dropped, and 27.6% (n = 8) mentioned that it had improved. The remaining students (n = 9, 31%) mentioned that they had not observed any change in GPA. Most of the students were aware of the medical use of stimulants (n = 24, 82.8%). The route of administration was mainly oral, as reported by 28 (96.6%) students who reported illicit use as shown in Table 5. 

**Table 5:** Information and awareness related to stimulant use N: number of students; GPA: grade point average

	Illicit use (N = 29)
First started using stimulant drugs	
First-year	5 (17.2%)
Second-year	9 (31.0%)
Third-year	8 (27.6%)
Fourth-year	4 (13.8%)
Fifth-year	3 (10.3%)
Taking stimulants produced the desired results	
No	7 (24.1%)
Sometimes	8 (27.6%)
Yes	14 (48.3%)
Observed GPA changes	
No	9 (31.0%)
Yes, dropped	12 (41.4%)
Yes, improved	8 (27.6%)
Quantity used per occasion, mg	
1–5	8 (27.6%)
6–10	10 (34.5%)
11–20	4 (13.8%)
21–30	5 (17.2%)
30–50	1 (3.45%)
More than 50	1 (3.45%)
Number of occasions used during study years	
1–10	9 (31.0%)
11–25	14 (48.3%)
26–50	1 (3.45%)
More than 50	5 (17.2%)
Number of occasions during previous year	
Never	1 (3.45%)
1–10	15 (51.7%)
11–25	9 (31.0%)
2650	2 (6.90%)
More than 50	2 (6.90%)
Route	
Intravenous	1 (3.45%)
Oral	28 (96.6%)
Aware of the medical use	
No	5 (17.2%)
Yes	24 (82.8%)

Irritability and headaches were the two most recorded side effects in both groups (67.64% and 64.70%, respectively). Remarkably, all the side effects showed equal prevalence between medical users and illicit users as shown in Table 6.

**Table 6:** Side effects of stimulant use across groups N: number of students

	Medical users	Illicit users	P-value
	N = 39	N = 29	
Irritability			.102
No	9 (23.1%)	13 (44.8%)	
Yes	30 (76.9%)	16 (55.2%)	
Headache			.706
No	15 (38.5%)	9 (31.0%)	
Yes	24 (61.5%)	20 (69.0%)	
Stomach ache			.106
No	14 (35.9%)	17 (58.6%)	
Yes	25 (64.1%)	12 (41.4%)	
Dizziness			.675
No	22 (56.4%)	14 (48.3%)	
Yes	17 (43.6%)	15 (51.7%)	
Difficulty getting along with friends			1.000
No	26 (66.7%)	20 (69.0%)	
Yes	13 (33.3%)	9 (31.0%)	
Becoming dependent on stimulants			1.000
No	24 (61.5%)	17 (58.6%)	
Yes	15 (38.5%)	12 (41.4%)	
Reduced appetite			.363
No	23 (59.0%)	13 (44.8%)	
Yes	16 (41.0%)	16 (55.2%)	
Sadness			.675
No	17 (43.6%)	15 (51.7%)	
Yes	22 (56.4%)	14 (48.3%)	
Nausea			1.000
No	38 (97.4%)	29 (100%)	
Yes	1 (2.56%)	0 (0.00%)	
I feel alone			1.000
No	38 (97.4%)	29 (100%)	
Yes	1 (2.56%)	0 (0.00%)	

**Figure 5:**
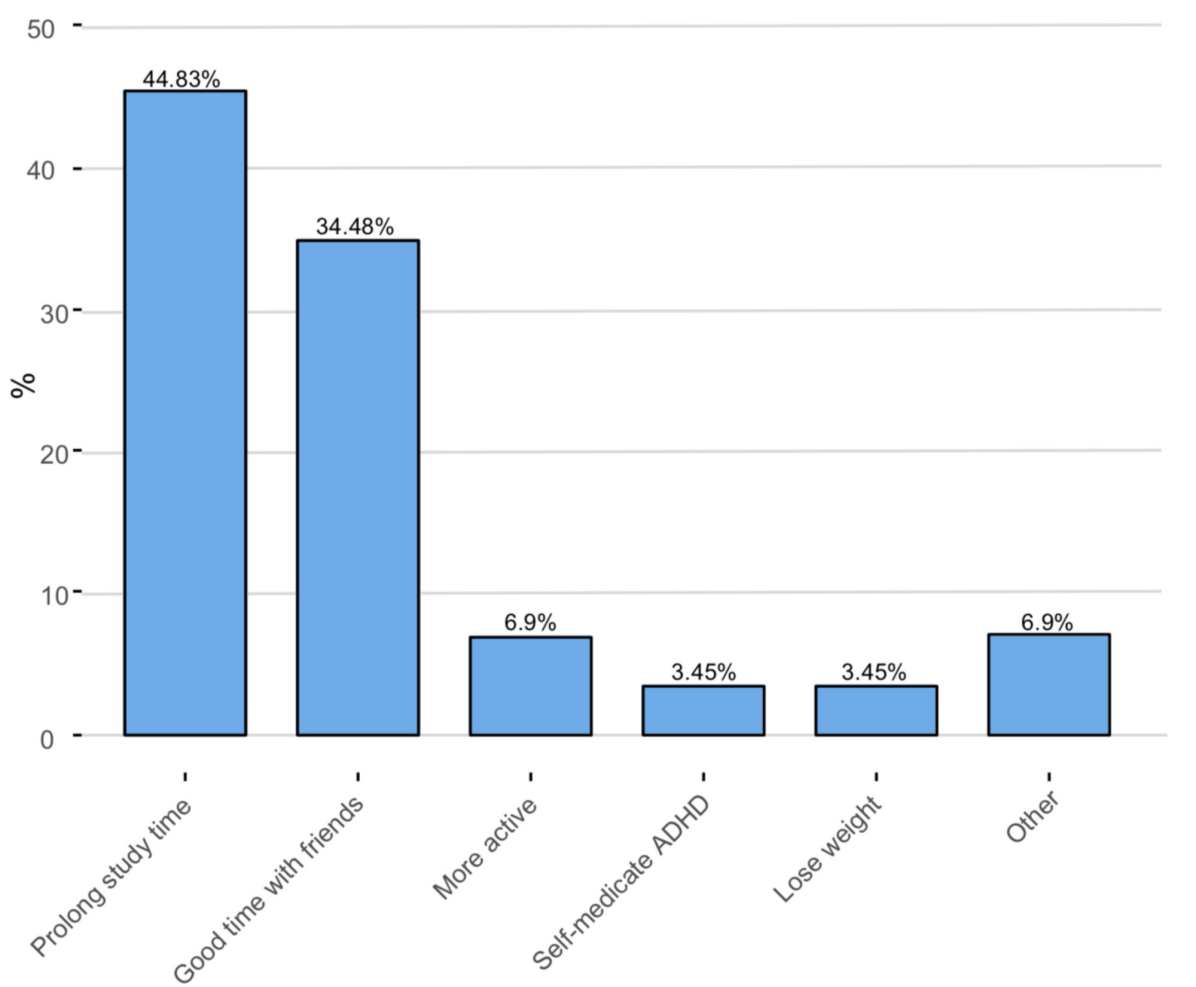
Reason for illicit use The Y-axis represents the percentage of illicit users of stimulants

The ability to prolong study time (n = 13, 44.83%) and having a good time with friends (n = 10, 34.48%) were the two most common reasons for illicit use. (Figure 5)

**Figure 6:**
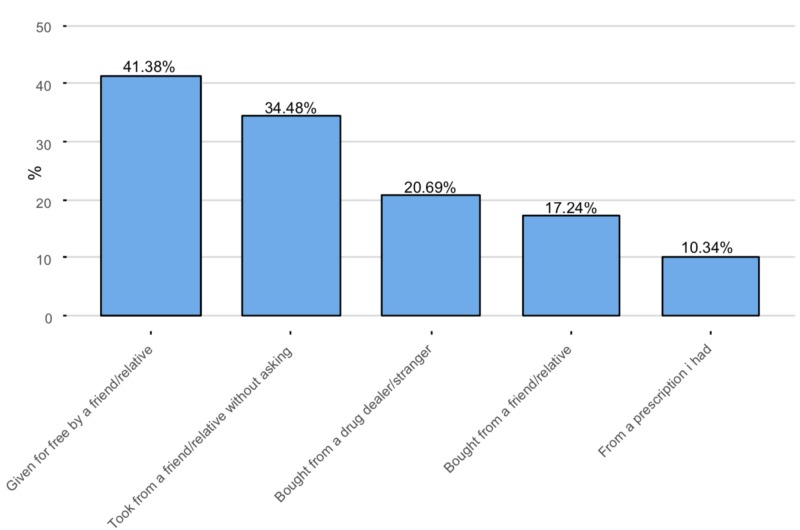
Source of stimulants The Y-axis represents the percentage of the illicit users of stimulants

Participants who reported illicit use mentioned that they were given it for free by a friend/relative (n = 12, 41.38%) or took it from them without asking (n = 10, 34.48%). (Figure 6).

The major concern reported by half of the students as shown was the need for stimulants to perform better (n = 14, 48.28%). The second most commonly reported concern was the availability of stimulants (n = 12, 41.38%), and the third was the dependency on stimulants (n = 11, 37.93%).

**Figure 7:**
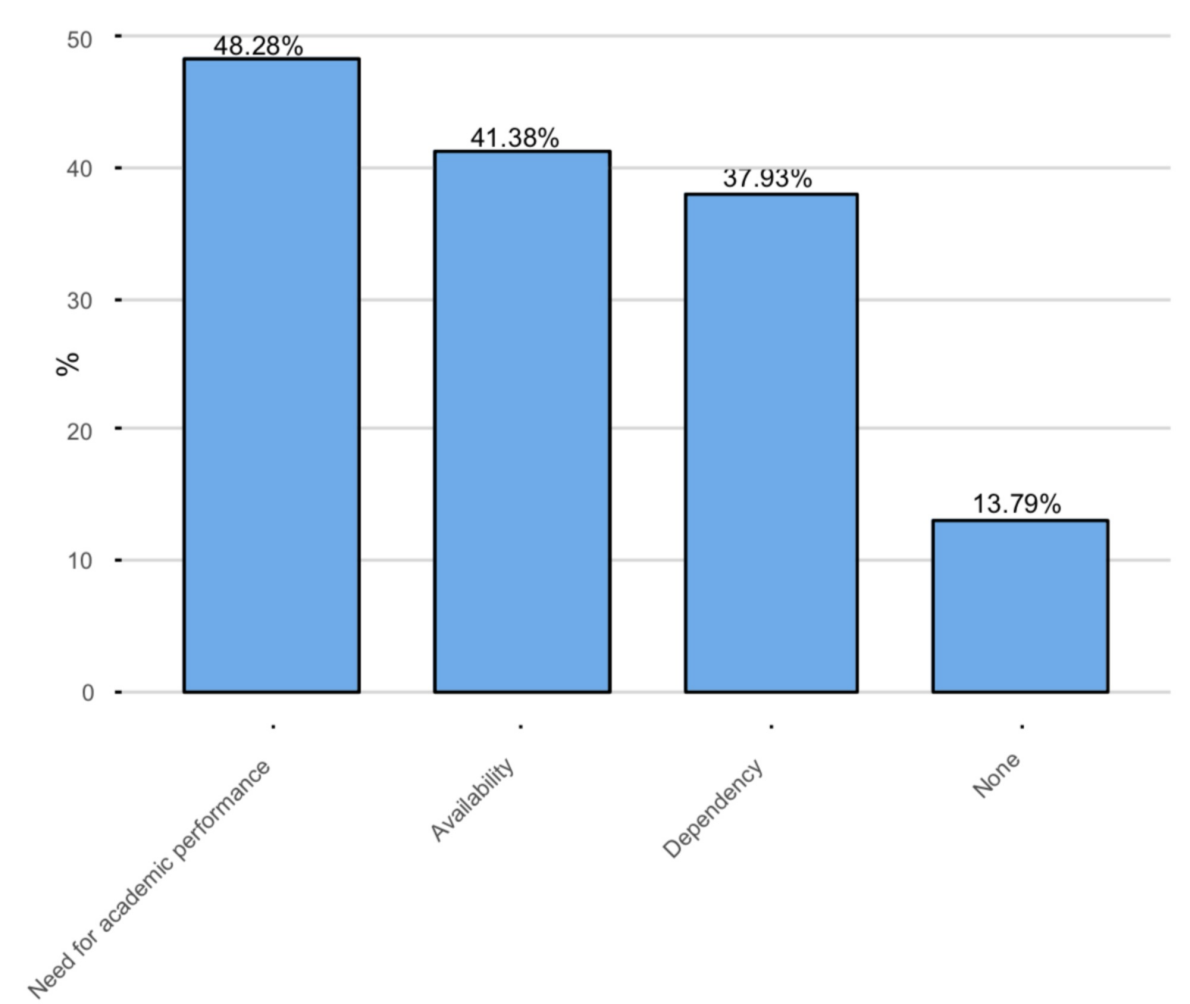
Concerns regarding the use of stimulants The Y-axis represents the percentage of the illicit users of stimulants

## Discussion

The current study aimed to investigate the prevalence of illicit use of stimulants among medical students in Riyadh by distributing an online survey to students of three government universities and included all years of the medical study. Of the 1,177 medical students that participated in our study, 68 (5.8%) admitted to using stimulant drugs; 39 (57.4%) were using the stimulants medically as they had been diagnosed with ADHD, whereas 29 (42.6%) had not been diagnosed with ADHD. Although the prevalence of illicit use of stimulant in this study (2.46%) was lower than that seen in some international surveys, it is almost comparable to that of college students in some other studies [4,7]; however, it is lower compared to the findings from other research conducted in smaller samples [5,9,10].

We noticed that as students progressed to a more senior level, there was a higher risk of using stimulants; more than half of the illicit users (58.6%) were in the fifth or final year. A study conducted in 2016 documented that there was a notable rise in the stress levels of medical students during the eighth semester of the fourth-year as students start to make the entry to internship/clerkship stage [11]; these students will be facing the most challenging period of their medical years due to the confusion of choosing a medical specialty, the difficulty of the Saudi Medical License Exam (SMLE), and the stiff competition they face in residency program entry. This may indicate that stress level plays a major role in the usage of non-medical stimulants.

Since GPA is one of the major concerns for all medical students as it affects their medical career, all students are ambitious about improving their academic performance to get higher GPAs. No matter what their current GPA was, these students were under continuous pressure to perform better academically. This assumption is supported by a study that found that performance pressure was one of the most common documented stress factors that medical students face [12]. In our study, illicit users were equally distributed throughout the different GPA levels. This shows that students are always under constant fear of getting low grades irrespective of their current GPAs. Similar to a previous study that looked at the association between ADHD symptoms and smoking, which concluded that the relationship was significantly positive [13], the current study found a high percentage of smokers among the medical users of stimulants compared to the non-users group (79.5% and 18.7%, respectively).

Adults with one risky behavior are highly susceptible to be engaged in other risky behaviors, as proven by one of the most reliable behavioral theories (Jessor’s Problem Behavior Theory) [14]. In our sample, the rate of smoking was remarkably high among illicit users. Additionally, taking stimulants from a friend or relative without asking is considered offensive but tended to be one of the most common sources of accessing the stimulants in our data. All of these actions can be considered bad behavior and support Jessor’s theory.

Regarding the motives for taking the stimulants, allowing students to choose only one main motive rather than choosing other secondary, less important motives helped us to identify the exact reason for the illicit use. The ability to prolong the study time (44.83%) was the main motive the students reported. When students were asked if taking the stimulant produced the desired result or not, about half of the students reported a positive impact (48.3%), whereas only (24.1%) were not getting their desired result. Reports show a higher frequency of taking stimulants is associated with higher rates of achieving the desired results. So, believing that taking stimulants will produce the desired effect will give the students more reasons to keep on using them. To find out is this true or not, and since the main motive was prolonging the study time, we asked the students about the observed changes in their GPAs. The results revealed a higher percentage of students reporting either a decrease or no change in their GPAs (41.4% and 31%, respectively).

In addition to the main objectives and demographic differences between the groups, we also assessed the route of administration of the stimulants, a point that has not been previously addressed in the literature. Only one student reported taking the stimulants intravenously; all other users reported an oral route of administration.

Limitations

This study has some limitations and these need to be taken into account when interpreting the results. The study aimed to collect data from all government universities in Riyadh, which should have involved Princess Nourah bint Abdulrahman University (PNU) as well. However, there was a concern as to whether including PNU in the collecting process would lead to a shift in the gender variable since PNU is an exclusively female university. Because of this, PNU was not included in our study. In the self-reported survey, a couple of questions relied on old memories, which may have introduced recall bias. The question regarding the number of missed days in the survey might have been answered inaccurately by the respondents. We could not reach a consensus about defining a missing day pertaining to all the participating universities, as each university maintains a unique schedule for the students, which may not match with other universities' schedules.

## Conclusions

Medical students always find themselves competing with their peers throughout their entire career. This may lead the students to use stimulant drugs illicitly. We hope that the present study would throw more light on the prevalence of illicit stimulant use among students in Riyadh. We urge the clinicians to be mindful of the potential to abuse the drugs they prescribe and to work on strategies to detect and minimize this abuse. We recommend that universities appoint specialists in this filed to educate the students about the adverse consequences of abusing the stimulants and to detect the abusers to counsel them.

Until further studies suggest otherwise, there is no evidence to show that medical students will completely achieve their desired outcomes by abusing illicit stimulants. Students need to be educated about other safe and legal methods for coping with burnout and stress.
